# Translation and evaluation of psychometric properties of the Dutch version of the Single Assessment and Numeric Evaluation Method (SANEM) in shoulder patients

**DOI:** 10.1186/s13018-019-1335-2

**Published:** 2019-09-05

**Authors:** Dieuwertje M. J. Theeuwen, Maria C. van der Steen, Inge F. M. Bonneux, Anouk M. E. Giesberts, Henk W. J. Koot, Max Reijman

**Affiliations:** 1 Orthopaedic Center Máxima, Máxima Medical Center, Ds. Theodor Fliednerstraat 1, 5631 BM Eindhoven, the Netherlands; 2 Department of Orthopaedic Surgery, Catharina Hospital, Michelangelolaan 2, 5623 EJ Eindhoven, the Netherlands

**Keywords:** SANEM, Shoulder, PROM, Translation, Validation, Construct validity, Reliability, Responsiveness

## Abstract

**Background:**

The Single Assessment Numeric Evaluation Method (SANEM) is a holistic patient-reported outcome measure (PROM) that includes all aspects involving the shoulder. It is simple and easy to administer. It consists of only one question, namely how would you rate your shoulder today as a percentage of normal (0 to 100% with 100% being normal)? The purpose of this study was to translate the SANEM in Dutch and to assess its construct validity, reliability, and responsiveness.

**Methods:**

The SANEM was translated into Dutch using forward and backward translation. Hypothesis testing was used to determine construct validity and responsiveness, 75% needed to be confirmed. Previous validated PROMs were used as comparator instrument for testing construct validity. Test-retest reliability (2-week interval), Standard Error of Measurement, and Smallest Detectable Change were calculated as reliability analyses. One year after baseline, we evaluated the responsiveness.

**Results:**

One hundred seven patients (55% women) with a mean age of 54 years were included. Of the hypotheses formed in advance to assess construct validity, 67% was confirmed, meaning there was no adequate construct validity and the SANEM cannot replace all other PROMs. With an intraclass correlation coefficient of 0.95, excellent test-retest reliability was found. Of the hypotheses formed in advance to evaluate the responsiveness, 75% was confirmed, indicating the SANEM has good responsiveness.

**Conclusion:**

Although the SANEM cannot replace all other PROMs, it is a reliable instrument to assess if a patients’ shoulder, regarding the whole shoulder, changes over time or stays unchanged.

**Level of evidence:**

Level II

## Background

Shoulder-related health issues are common in the general population. A systematic review showed an average lifetime prevalence up to 67% [1]. Common shoulder-related problems that are observed at the outpatient department are subacromial pain syndrome, rotator cuff tears, frozen shoulder, instability, and osteoarthritis. There are multiple methods available to measure the clinical condition of the shoulder and to assess the outcome of treatment. Some are clinician-based and involve a physical examination while others are patient-based in which patients respond to the questionnaires themselves [2–8].

Patient-based questionnaires can be used as patient-reported outcome measures (PROMs). PROMs are developed to measure a patient’s perception of their functional status and wellbeing. They are becoming more and more important in evaluating the treatment of patients with all kind of diseases. Some PROMs evaluate patient’s perception of their overall health and quality of life while others are disease-specific or body part-specific PROMs [9–11].

Most PROMs for evaluating the shoulder focus on a specific aspect of the shoulder, for example, pain, functional status, or stability [3, 9, 10, 12]. To obtain a clear picture of the overall health status of the shoulder, multiple time-consuming questionnaires have to be fulfilled. The disadvantage of using multiple PROMs is that it is time-consuming, and in our clinic, patients experience it as a burden. A more holistic measurement that includes all aspects of health involving the shoulder is needed.

The preferred measurement tool should be simple and easy to administer for patients of every educational level. The Single Assessment Numeric Evaluation Method (SANEM) is such a measurement tool. This easy to administer PROM consists of only one question, namely how would you rate your shoulder today as a percentage of normal (0 to 100% with 100% being normal)? It was already validated in the English language. Once in a population consisting out of 163 United States Military Academy cadets after shoulder surgery and once in a population of 441 patients who underwent an operative treatment for rotator cuff repair, arthroplasty of stabilization for recurrent anterior shoulder dislocation [13, 14]. The aim of the current study was to translate the SANEM into the Dutch language. Thereafter, we aimed to evaluate the psychometric properties of the Dutch version of the SANEM in patients with shoulder complains, in terms of validity, reliability, and responsiveness.

## Methods

### Translation procedure

The translation procedure was done according to the guidelines for the process of cross-cultural adaptation by Beaton et al. [15]. First, a forward translation was performed. Two native Dutch speakers with adequate knowledge of the English language independently translated the original English version. One translator had expertise on the questionnaire under study, and the other translator was naïve about the topic of the study. Both translators had a medical background. In a consensus meeting between the two translators, differences were identified and resolved. This led to the first preliminary version of the SANEM.

Subsequently, two native English speakers with adequate knowledge of the Dutch language, independently and totally blinded to the original version, performed the backward translation. Both translators were no experts on the construct to be measured and had no medical background. The primary purpose of this step was that the translation is reflecting the same item content as the original version.

Finally, after the backward translation, the second consensus meeting involved the four translators. All translations were reviewed and discrepancies were discussed. This led to the pre-final version of the SANEM. This pre-final version was evaluated by a department at our hospital, who are experienced in patient questionnaires. They made no further alterations to the pre-final version.

This version was tested in a small pilot study to assess the comprehensibility of the questionnaire. Fifteen patients of a variety of educational backgrounds, who visited the outpatient department due to a shoulder-related problem, were interviewed. They were asked if they understood the question, if they had any suggestions for improvement, and if they could describe the meaning of the question in their own words. These patients were not included in the validation study.

In the last consensus meeting with all four translators, the feedback of the patients of the pilot study and all written reports were discussed, and no large adjustments were made. This led to the final Dutch version of the SANEM: “Hoe zou u uw schouder vandaag beoordelen op een schaal van 0% tot 100% wanneer 100% normaal is?”

### Participants

Patients were consecutively recruited between October 2016 and January 2017 from the outpatient department of orthopedics. Approval for this study was obtained from the local ethical committee, and written informed consent was obtained from all participants. The goal of this study was to include at least 100 participants, as is advised by the Consensus-based Standards for the Selection of health Measurement INstruments (COSMIN) criteria to assess cross-cultural validity [16]. Patients were eligible if they were 18 years or older and visited the outpatient department with a shoulder-related problem. Exclusion criteria were insufficient knowledge of the Dutch language, patients who recently underwent an operation and as a result a physical examination could not be performed and patients who could not respond by e-mail. Patients’ characteristics at baseline are described in Table 1.

**Table 1:** Baseline characteristics

Total participants	107
Female, *n* (%)	55 (51.4)
Mean age, years (SD)	54.4 (12.1)
Diagnosis, *n* (%)	
Rotator cuff tears	36 (33.6)
Frozen shoulder	25 (23.4)
SAPS	13 (12.1)
Osteoarthritis	9 (8.4)
Labrum defect	5 (4.7)
Instability	4 (3.7)
Fracture	2 (1.9)
Atypical/other complains	13 (12.2)

*SD* standard deviation, *SAPS* subacromial pain syndrome

### Study procedure

At baseline (T0), included patients had to complete the Dutch versions of the Numerica (Pain) Rating Scale (NRS-pain), EuroQol five-dimensions scale (EQ-5D), Simple Shoulder Test (SST), Oxford Shoulder Score (OSS), or Oxford shoulder Instability Score (OSIS) if they had an instability-related problem, Constant Score (CS), and the SANEM at the outpatient department. These results were used to measure the construct validity of the SANEM.

Six months after all participants were included (T1), they received an e-mail to complete the SANEM at home. Two weeks later (T2), they again received an e-mail to fulfill the SANEM together with two anchor questions again at home. T1 and T2 were used to assess the reliability of the SANEM.

One year after baseline (T3), patients received the last e-mail to complete the SANEM together with two anchor questions. This was used to assess the responsiveness of the SANEM.

### SANEM

The English version of the SANEM is determined by asking the patient the following question: How would you rate your shoulder today as a percentage of normal (0 to 100% with 100% being normal)? [14]. In the English literature, this numeric evaluation is also known as the Subjective Shoulder Value (SSV) [13].

The SANEM is easy to administer and quick measurement tool which represents the direct view of the patient on its shoulder. It is not a shoulder-specific or disease-specific measurement. The advantage is its simplicity and applicability to all kind of shoulder-related problems.

In the English literature, it is validated against the American Shoulder and Elbow Surgeon Score (ASESS), the Rowe score, and the Constant score [13, 14]. Both studies found a high correlation between the SANEM and the other scores and concluded that the SANEM should be considered as a convenient adjunct to clinician-reported scoring systems.

### Comparative questionnaires

#### Constant score (CS)

The Constant score, also known as the Constant-Murley score, is a shoulder-specific measurement. It contains four subscales: pain score, functional assessment, range of motion (flexion, lateral elevation, internal rotation, and external rotation), and strength. The maximum score is 100 which represents an excellent shoulder, and the lowest score is 0 which represents a poor shoulder. This score records individual parameters and provides an overall clinical functional assessment [3]. The Constant score is a clinician-reported measurement which includes a physical examination and thus can only be applied in clinical settings. In the English language, it has been validated for patients with several shoulder-related problems such as rotator cuff tears, osteoarthritis, and instability. It is not validated in the Dutch language but is used in almost every language without official translations since it is reported by the clinician [8].

#### Simple Shoulder Test (SST)

The Simple Shoulder Test is a shoulder-specific PROM. It was developed in the USA to measure functional limitations in patients with common shoulder problems [12]. It contains 12 simple questions about activities. For each question, the patient indicates if he or she is able to perform the activity or not. The maximum score is 12, which represents an excellent shoulder function and the lowest score is 0 which represents a poor shoulder function. The SST was validated in the Dutch language by Kampen et al. [5].

#### Oxford Shoulder Instability Score (OSIS)

The Oxford Shoulder Instability Score is a shoulder-specific measurement which is also disease-specific for patients with instability problems. It was developed in the UK by Dawson et al. in order to assess the outcome of a treatment for shoulder instability [11]. The OSIS contains 12 questions about pain and shoulder function with 5 responses for each question. Answers are scored from 0 to 4. A total score of 0 represents the most impaired shoulder and 48 represents the least impaired shoulder. The OSIS was validated in the Dutch language by van der Linde et al. [6].

#### Oxford Shoulder Score (OSS)

The Oxford Shoulder Score is a shoulder-specific measurement. It was developed in the UK by Dawson et al. for patients undergoing shoulder surgery and the questions regarded pain and shoulder function [10]. Just like the OSIS, it also contains 12 questions with 5 responses for each question where answers are scored from 0 to 4. A total score of 0 represents the most impaired shoulder and 48 the least impaired shoulder. The OSS was validated in the Dutch language by Berendes et al. [2].

#### EQ-5D

The EQ-5D is a five-dimensional questionnaire to measure the generic health status of the patient. The five dimensions are mobility, self-care, usual activities, pain/discomfort, and anxiety/depression. Each dimension consists of 3 answers, namely no problems, some problems, or not being able to accomplish. In addition to the five dimensions, it also contains a visual analog scale (VAS) for overall health status. The minimum score is − 0.21 and represents the worst possible general health status. The maximum score is 1 and represents the best possible health status [4]. The EQ-5D has been translated into most major languages, including the Dutch language [17].

#### NRS-pain

The NRS-pain is used to assess pain intensity during rest and during movement. Patients are asked to indicate the level of pain they experience by reporting a number from 0 to 10. Zero represents no pain and 10 represents the worst imaginable pain. The NRS pain is a valid questionnaire in adult patients with musculoskeletal-related problems [18, 19]. In this study, two NRS-pain questions were asked. One concerns a situation where the patient is resting and one where the patient is using the shoulder for at least minimally intensive activities.

### Assessment of psychometric properties

#### Construct validity

Validity refers to the degree an instrument measures what it claims to measure. In shoulder patients, there is no golden standard to assess the clinical status of the shoulder. Therefore, the construct validity is measured by determining the correlation between the SANEM and other instruments that aim to evaluate the same construct and are already used or validated in the Dutch language. Before starting the study, hypotheses about the expected correlations were formed (see Table 2). At T0, all participants fulfilled the SANEM together with 5 other questionnaires. Pearson’s correlation coefficient was used to calculate the correlations. We classified the correlations into three categories: high correlation (*r* = > 0.5), moderate correlation (*r* = 0.3–0.5), and low correlation (*r* = < 0.3). As described in the COSMIN criteria, we considered construct validity to be of an adequate standard when at least 75% of the hypotheses were confirmed [16].

**Table 2:** Construct validity, hypotheses, and confirmation

Hypotheses	Pearson’s correlation (95% confidence interval)	Hypothesis confirmed
1. The correlation between the SANEM and the CS is > 0.50 (high).	0.52 (0.35–0.65)	Yes
2. The correlation between the SANEM and the SST is > 0.50 (high).	0.48 (0.30–0.62)	No
3. The correlation between the SANEM and the OSS/OSIS is > 0.50 (high).	0.59 (0.46–0.70)	Yes
4. The correlation between the SANEM and the NRS-pain-rest is ≤ − 0.50 (high).	− 0.41 (− 0.58 to − 0.22)	No
5. The correlation between the SANEM and the NRS-pain active is ≤ − 0.50 (high).	− 0.50 (− 0.64 to − 0.33)	Yes
6. The correlation between the SANEM and the EQ-5D is 0.30–0.50 (moderate).	0.30 (0.12–0.45)	Yes
7. The correlation between the SANEM and the OSS/OSIS is > 0.1 higher than that between the SANEM and the SST.		Yes
8. The correlation between the SANEM and the OSS/OSIS is > 0.1 higher than that between the SANEM and the NRS-pain rest.		Yes
9. The correlation between the SANEM and the OSS/OSIS is > 0.1 higher than that between the SANEM and the NRS-pain active.		No
**Percentage of hypotheses confirmed**		**67%**

*SANEM* Single Assessment Numeric Evaluation Method, *CS* Constant score, *SST* simple shoulder test, *OSS* Oxford Shoulder Score, *OSIS* Oxford Shoulder Instability Score, *NRS* Numeric Rating Scale, *EQ-5D* EuroQol five-dimension scale

#### Reliability

##### Test-retest reliability

The test-retest reliability gives information about the variance in scores taken by the same measurement instrument under the same conditions. It was tested by letting patients answer the SANEM at T1 and again at T2, both times together with two anchor questions about pain and function. At T1 and T2, we considered it important that the questions are answered under the same conditions. This is why both time participants fulfill the questionnaire at home by responding to an e-mail. The time interval between T1 and T2 must be sufficiently long to prevent patients from remembering the previous answer they gave at T1 but short enough to avoid any changes in circumstances regarding pain and function. In this study, 2 weeks were considered to be an adequate time interval [20]. For this calculation, we only used the participants who replied to the anchor question that there were no of little differences between T1 and T2 regarding pain and function. The intraclass correlation coefficient (ICC) between T1 and T2 was calculated. An ICC > 0.75 was considered an excellent correlation as advised by the COSMIN criteria [16]. Analysis was performed using a two-way mixed model with an absolute agreement.

##### Measurement error

Measurement error tells us something about the possible difference between the score obtained and the actual score. It was evaluated by calculating the standard error of measurement (SEM) and the smallest detectable change (SDC). The SEM can be implied as the standard deviation of repeated test scores from one single patient if the circumstances do not change. It gives information about the precision of one score. Low levels of SEM indicate a high level of score accuracy. It is calculated using the following formula: SEM = (SD difference/√2). Two stands for the 2 measurement repetitions we performed in this study to assess reliability (T1 and T2). The SDC is calculated using the formula: SDC = 1.96 × √2 × SEM [20]. It represents the smallest change in a patient’s score that is not the result of measurement error. A low SDC tells us that a little difference in the clinical state of the patient can be measured with this instrument without measurement error.

#### Responsiveness

Responsiveness is defined as “the ability of an instrument to detect change over time in the construct to be measured”. In case of accurate responsiveness, if the score of a patient changes, the score of the measurement instrument changes with it.

We evaluated the responsiveness by comparing the change of the SANEM between T0 and T3. First, we calculated the effect size (ES) as follows: (mean T3–mean baseline scale)/SD of baseline scale. Furthermore, we calculated the standardized response mean (SRM) as follows: (mean T3–mean baseline scale)/SD of change in scales. All changes between T0 and T3 were converted to absolute values. Table 4 shows the hypotheses that were formed in advance. Hypotheses were formulated separately for three groups, namely patients who reported to the anchor question that there was a difference in pain AND function compared to baseline, patients who reported there was a difference in pain OR function, and patients who reported there was no difference in both pain AND function. There were 7 answer options to the anchor questions, namely very much improved/impaired, much improved/impaired, a little improved/impaired, or no difference. Patients who answered to the anchor question that there was a little improvement or impairment were also added to the “no difference group”. An ES/SRM of ≤ 0.2 was interpreted as no difference, ≥ 0.2 as small, ≥ 0.4 as medium, and ≥ 0.8 as large. Responsiveness was scored as adequate if minimally 75% of the hypotheses were confirmed.

## Results

A total of 107 patients who visited the outpatient clinic with a shoulder-related problem, fulfilled to the criteria, and were willing to participate were recruited. Baseline characteristics are shown in Table 1.

### Construct validity

Table 2 shows the hypotheses that were formed in advance of the study and the correlations that were found between the SANEM and the comparative measurement instruments. It also represents if the formulated hypotheses are confirmed. The table shows that 67% of our hypotheses were confirmed, which is less than the 75% we needed for adequate construct validity.

### Reliability

For determining the reliability, 56 patients replied to the anchor question that there were no differences between T1 and T2 regarding pain and function. Table 3 shows the score of the test-retest reliability and the aspects of measurement error.

**Table 3:** Reliability analysis (*n* = 56)

Mean score T1	Mean score T2	Mean difference (T1-T2)	ICC (95% CI)	SEM	SDC
71.2 (± 21.0)	72.3 (± 20.5)	5.3 (± 7.5)	0.95 (0.91–0.97)	5.3	14.6

Data are presented as mean and corresponding standard deviation or reported otherwise as mentioned. T1: 6 months after T0, T2: 2 weeks after T1

*ICC* intraclass correlation coefficient, *SEM* standard error of measurement, *SDC* smallest detectable change, *CI* confidential interval

### Responsiveness

For determining the responsiveness, there was a loss to follow up of 3 patients. Forty-nine patients replied to the anchor question that there was a difference in pain and function, 14 replied that there was a difference in pain or function, and 41 replied that there was no difference. Table 4 shows the hypotheses that were formed in advance and their confirmation. Six out of 8 hypotheses were confirmed indicating that the SANEM has an adequate responsiveness.

**Table 4:** Responsiveness: hypotheses and confirmation

Hypotheses	ES/SRM	Hypothesis confirmed
1. The ES in the group of patients who reported there was a difference between T0 and T3 in pain **and** function is expected to be ≥ 0.8.	1.24	Yes
2. The SRM in the group of patients who reported there was a difference between T0 and T3 in pain **and** function is expected to be ≥ 0.8.	1.39	Yes
3. The ES in the group of patients who reported there was a difference between T0 and T3 in pain **or** function is expected to be ≥ 0.4.	1.02	Yes
4. The SRM in the group of patients who reported there was a difference between T0 and T3 in pain **or** function is expected to be ≥ 0.4.	1.59	Yes
5. The ES in the group of patients who reported there was no difference between T0 and T3 in pain **and** function is expected to be ≤ 0.2.	0.33	No
6. The SRM in the group of patients who reported there was no difference between T0 **and** T3 in pain and function is expected to be ≤ 0.2.	0.30	No
7. The ES in the group of patients who reported there was a difference between T0 and T3 in pain **and** function is expected to be ≥ 0.2 larger than the ES in the group of patients who reported there was a difference in pain **or** function between T0 and T3.		Yes
8. The SRM in the group of patients who reported there was a difference between T0 and T3 in pain **and** function is expected to be ≥ 0.2 larger than the SRM in the group of patients who reported there was a difference in pain **or** function between T0 and T3.		Yes
**Percentage of hypotheses confirmed**		**75%**

*ES* effect size, *SRM* standardized response mean

## Discussion

Assessment of clinical outcomes with PROMs is increasingly important in the evaluation of patients [9]. The SANEM measures the direct view of the patient regarding the status of the whole shoulder. In the current study, we translated the SANEM into the Dutch language and evaluated the psychometric properties in a population of patients with shoulder-related problems. The Dutch version of the SANEM showed adequate reliability and responsiveness; however, construct validity was not confirmed.

Evaluating the construct validity, only 67% of our hypotheses were confirmed, which is less than the 75% needed for an adequate construct validity [15]. We hypothesized that the SANEM would correlate high with the applied other shoulder-specific PROMs and physician-reported outcome measures, as the SANEM is intended to measure all aspects of the shoulder. In line with previous studies, the SANEM showed a high correlation with CS [13, 14]. The OSS and OSIS also correlated high with the SANEM, another expected result since they both measure function and pain [10, 11]. A moderate correlation between the SST and the SANEM was found. A possible explanation why the SST correlates lower with the SANEM compared to the other shoulder-specific PROMs is that the SST only measures function and not pain, while the SANEM measures the whole status of the shoulder [12]. The correlation between the SANEM and the NRS-pain was high when patients were “active” and moderate instead of high when patients were “resting.” When formulating the hypotheses, we assumed that when patients were asked if they experience pain when they are resting, they would also take into account the pain they experience during the night. Pain at night is a common problem for patients with several shoulder-related problems and a large part of the patients considers this to be the main problem they experience. Furthermore, we hypothesized that the correlation between the SANEM and the OSS was 0.1 higher than between the SANEM and the NRS-pain active. The difference found between these two correlations was 0.09. Although this difference is very close to what we hypothesized, we should consider that when evaluating the whole shoulder, patients seem to attain more value to the pain they experience than we expected. Overall, although 67% of the hypotheses were confirmed and multiple hypotheses were nearly confirmed, the construct validity of the Dutch version of the SANEM cannot be evaluated as adequate. This suggests that the SANEM cannot replace all other Dutch PROMs and physician-reported outcome measures.

Test-retest reliability, measurement error, and responsiveness were not previously described for the SANEM. When determining the test-retest reliability, we found an excellent ICC. Assessing the rest-retest reliability, it is important that during both measurement moments, circumstances are similar [16]. We therefore explicitly chose to let the participants fulfill the SANEM both times at home only together with two anchor questions and no other questionnaires. The SEM was found to be 5.3. There is little literature available on how to interpret the SEM and when it can be considered as adequate. However, low levels of SEM indicate high levels of score precision. The SDC found was 14.6. This means that between two measurement moments, patients need to score a difference of at least 14.6% in order to assume that the difference is not a result of measurement error. Overall, the Dutch version of the SANEM showed excellent test-retest reliability and the SDC was determined.

In the assessment of responsiveness, 75% of the in advance formed hypothesis were confirmed, indicating that the Dutch version of the SANEM has an adequate responsiveness. Considering the results of the reliability and responsiveness assessment, we conclude that the SANEM is a useful measurement instrument to use in shoulder patients between two consultations.

Williams et al. were the first to assess the validity of the English version of the SANEM in 163 patients with shoulder instability [14]. Gilbert et al. also evaluated an English numeric evaluation scale for the shoulder and named it the “Subjective Shoulder Value (SSV)”; they only included patients who underwent surgery [13]. Both studies found moderate to high correlations between the SANEM/SSV and other rating scores, unfortunately without formulating hypothesis in advance. Reliability and responsiveness were not described. Both studies concluded that the SANEM/SSV can be used as an additional tool when time and resources are limited but cannot replace other measurement instruments. There were no previous studies that determined the correlation between the SANEM and the other PROMs we used in the current study.

The main advantage of our study is that it is the first assessment of reliability and responsiveness of the SANEM. Another important strength of this study is that during the whole procedure COSMIN guidelines regarding validation of a PROM were accurately followed. Hypotheses were formed in advance to assess construct validity and responsiveness. We aimed to form an adequate number of hypothesis despite the lack of consensus or guidelines what can be considered as an adequate number. Another limitation might be that in the current assessment of construct validity and responsiveness, all hypotheses count equally despite that some can be found more important and/or elicit a stronger correlation. As previously used comparative instruments were not validated in the Dutch language, we were forced to use other PROMs than the studies that validated the English version of the SANEM [13, 14]. This might influence the comparability with previous literature, but enhances the measurement of validity in a Dutch population. As mentioned before, this is the first assessment of psychometric properties of the SANEM. Therefore, unfortunately, no comparison can be made to psychometric properties of the SANEM validated in the English language.

## Conclusion

A preferred measurement tool for assessing the shoulder should be simple and easy to administer for patients of every educational level. The Single Assessment Numeric Evaluation Method (SANEM) is such a measurement tool. We aimed to translate the SANEM and evaluate the psychometric properties of the Dutch version of the SANEM in patients with shoulder complains, in terms of validity, reliability, and responsiveness. Our results show that the SANEM by itself cannot replace other validated measurement instruments used to evaluate the shoulder. However, when time and resources are limited, our results indicate that the SANEM is a useful and reliable instrument to assess if between multiple consultations a patients’ shoulder, regarding the whole shoulder, changes over time or stays unchanged. Table 5 gives an overview of the results of this study.

**Table 5:** Summary results

Psychometric property	Value	Adequate
Construct validity (hypothesis confirmed)	67%	No
Reliability		
ICC	0.95%	Yes
SEM	5.28	
SCD	14.64	
Responsiveness (hypothesis confirmed)	75%	Yes

*ICC* intraclass correlation coefficient, *SEM* standard error of measurement, *SDC* smallest detectable change

## Data Availability

The datasets used and/or analyzed during the current study are available from the corresponding author on reasonable request.

## Abbreviations

ASESS: American Shoulder and Elbow Surgeon Score
CI: Confidential interval
COSMIN: Consensus-based Standards for the Selection of health Measurement INstruments
CS: Constant score
EQ-5D: EuroQol five-dimensions scale
ES: Effect size
ICC: Intraclass correlation coefficient
NRS: Numeric Rating Scale
OSIS: Oxford Shoulder Instability Score
OSS: Oxford Shoulder Score
PROM: Patient-reported outcome measure
SANEM: Single Assessment and Numeric Evaluation Method
SAPS: Subacromial pain syndrome
SD: Standard deviation
SDC: Smallest detectable change
SEM: Standard error of measurement
SRM: Standardized response mean
SST: Simple Shoulder Test
SSV: Subjective shoulder value
VAS: Visual analog scale

## References

[1] Luime JJ, Koes BW, Hendriksen IJ, Burdorf A, Verhagen AP, Miedema HS (2004). Prevalence and incidence of shoulder pain in the general population: a systematic review. Scand J Rheumatol.

[2] Berendes T, Pilot P, Willems J, Verburg H, te Slaa R (2010). Validation of the Dutch version of the Oxford Shoulder Score. J Shoulder Elb Surg.

[3] Constant CR, Murley AH (1987). A clinical method of functional assessment of the shoulder. Clin Orthop Relat Res.

[4] EuroQol Group (1990). EuroQol--a new facility for the measurement of health-related quality of life. Health Policy.

[5] Van Kampen D, van Beers L, Sholtes V, Terwee C, Willems W (2012). Validation of the Dutch version of the Simple Shoulder Test. J Shoulder Elb Surg.

[6] Van der Linde J, van Kampen D, van Beers L, van Deurzen D, Terwee C, Willems J (2015). The Oxford Shoulder Instability Score: validation in Dutch and first-time assessment of its smallest detectable change. J Orthop Surg Res.

[7] Rowe CR, Patel D, Southmard WW (1978). The Bankart procedure: a long-term end-result study. J Bone Joint Surg Am.

[8] Roy JS, MacDermid JC, Woodhouse LJ (2010). A systematic review of the psychometric properties of the Constant-Murley score. J Shoulder Elb Surg.

[9] Dawson J, Doll H, Fitzpatrick R, Jenkinson C, Carr A (2010). The routine use of patient reported outcome measures in healthcare settings. BMJ.

[10] Dawson J, Fitzpatrick R, Carr A (1996). Questionnaire on the perceptions of patients about shoulder surgery. J Bone Joint Surg (Br).

[11] Dawson J, Fitzpatrick R, Carr A (1999). The assessment of shoulder instability. The development and validation of a questionnaire. J Bone Joint Surg (Br).

[12] Lippitt SB, Harryman DT, Matsen FA (1993). A practical tool for evaluating function: the Simple Shoulder Test. The shoulder: a balance of mobility and stability.

[13] Gilbart M, Gerber C (2007). Comparison of the subjective shoulder value and the Constant score. J Shoulder Elb Surg.

[14] Williams GN, Gangel TJ, Arciero RA, Uhorchak JM, Taylor DC (1999). Comparison of the single assessment numeric evaluation method and two shoulder rating scales, outcomes and measures after shoulder surgery. Am J Sports Med.

[15] Beaton DE, Bombardier C, Guillemin F, Ferraz MB (2000). Guidelines for the process of cross-cultural adaptation of self-report measures. Spine.

[16] Terwee CB, Mokkink LB, Knol DL, Ostelo RW, Bouter LM, de Vet HC (2012). Rating the methodological quality in systematic reviews of studies on measurement properties: a scoring system for the COSMIN checklist. Qual Life Res.

[17] Rabin R, Charro F (2001). EQ-SD: a measure of health status from the EuroQol group. Ann Med.

[18] Hawker G, Mian S, Kendzerska T, French M (2011). Measures of adult pain: Visual Analog Scale for Pain (VAS Pain), Numeric Rating Scale for Pain (NRS Pain), McGill Pain Questionnaire (MPQ), Short-Form McGill Pain Questionnaire (SF-MPQ), Chronic Pain Grade Scale (CPGS), Short Form-36 Bodily Pain Scale (SF-36 BPS), and Measure of Intermittent and Constant Osteoarthritis Pain (ICOAP). Arthritis Care Res.

[19] Williamson A, Hoggart B (2005). Pain: a review of three commonly used pain rating scales. J Clin Nurs.

[20] De Vet HCW, Terwee CB (2011). Measurement in medicine.

